# Gene finding in metatranscriptomic sequences

**DOI:** 10.1186/1471-2105-15-S9-S8

**Published:** 2014-09-10

**Authors:** Wazim Mohammed Ismail, Yuzhen Ye, Haixu Tang

**Affiliations:** 1 School of Informatics and Computing, Indiana University, 150 S. Woodlawn Avenue, IN 47401 Bloomington, USA

**Keywords:** Metatranscriptomics, Gene finding, Hidden Markov Model, Operons, Antisense RNA (asRNA)

## Abstract

**Background:**

Metatranscriptomic sequencing is a highly sensitive bioassay of functional activity in a microbial community, providing complementary information to the metagenomic sequencing of the community. The acquisition of the metatranscriptomic sequences will enable us to refine the annotations of the metagenomes, and to study the gene activities and their regulation in complex microbial communities and their dynamics.

**Results:**

In this paper, we present TransGeneScan, a software tool for finding genes in assembled transcripts from metatranscriptomic sequences. By incorporating several features of metatranscriptomic sequencing, including strand-specificity, short intergenic regions, and putative antisense transcripts into a Hidden Markov Model, TranGeneScan can predict a sense transcript containing one or multiple genes (in an operon) or an antisense transcript.

**Conclusion:**

We tested TransGeneScan on a mock metatranscriptomic data set containing three known bacterial genomes. The results showed that TranGeneScan performs better than metagenomic gene finders (MetaGeneMark and FragGeneScan) on predicting protein coding genes in assembled transcripts, and achieves comparable or even higher accuracy than gene finders for microbial genomes (Glimmer and GeneMark). These results imply, with the assistance of metatranscriptomic sequencing, we can obtain a broad and precise picture about the genes (and their functions) in a microbial community.

**Availability:**

TransGeneScan is available as open-source software on SourceForge at https://sourceforge.net/projects/transgenescan/.

## Background

Fueled by rapid advances of sequencing techniques [1], culture-independent community-wide methods (known as the *metagenomics *approach) have been commonly used to study the microbial community in different environments, such as in soil [2], ocean [3], freshwater [4] and human-associated communities [5-7]. Large scale surveys of human microbiomes through the Human Microbiome Project (HMP) [6,7] and the MetaHit Project [5] have provided great resources for studying the diversity and complexity of human microbiomes and their potential impacts on human health and diseases. Comparison of human gut microbiomes of individuals with type II diabetes (T2D) against those of normal controls have revealed important taxonomic and functional differences of the microbial communities that might be correlated with T2D [8-10].

While metagenomic sequencing revealed important functional properties encoded in microbial communities, characterization of these properties requires direct analysis of the active component of the metagenome, through meta-omic techniques such as metatranscriptomics or metaproteomics. Bacteria have low inventories of short-lived mRNAs so that fluctuations in their mRNAs pools provide a highly sensitive bioassay for environmental signals (e.g., the concentrations of dissolved organic carbon [11] and pollutant concentrations [12]) that are relevant to microbes [13], and the collective interaction among microbial organisms in response to the changes of their environment ( e.g., the health condition of the host of human microbiome [14]).

In a common metatranscriptomic study (using the *RNA-seq *protocol), total RNA is first isolated from the sample and structural RNAs are then removed to enrich for mRNA, which is then reverse transcribed into cDNA subject to DNA sequencing using next generation sequencing (NGS) platforms like Illumina sequencers [15]. Metatranscriptomic data indicate which of the genes encoded in a metagenome are actually transcribed, and which of the potential metabolic pathways are active (and the level of their activities) on the basis of their transcriptions within a microbial community under certain environmental condition. HMP II, the second phase of the Human Microbiome Project (HMP) plans to generate coherent meta-omic (metagenomic, metatranscriptomic and metaproteomic) datasets acquired from the same cohorts of healthy human subjects and patients with certain diseases (including diabetes, Crohn's disease and ulcerative colitis).

In addition to elucidating functional characteristics of microbial communities, metatranscriptomic data provides valuable information for accurate annotations of genes and studies of their regulation in the community that are complementary to metagenomic sequencing. Metatranscriptomic sequences can be assembled into transcripts, each encoding one or more genes that are transcribed together (in the same direction). In the latter case (known as *operons*), the intergenic regions between coding genes are relatively short. In comparison, metagenomic sequences are assembled into contigs of genomic segments, many of which may contain long non-coding intergenic regions. In addition, current metatranscriptomic studies adopt the stranded RNA-seq protocol [15]; as a result, the genes should be encoded on the positive strand of the assembled transcripts in metatranscriptomic sequences, while in metagenomic sequences, genes can be encoded in either strand. These features of metatranscriptomic data can significantly improve the gene prediction accuracy in metatranscriptomic sequences, and thus should be incorporated into gene predictors specifically designed for metatranscriptomic data. Furthermore, because of the strand-specificity in metatranscriptomic data, antisense RNAs (asRNAs), which are encoded on the DNA strand opposite to a protein coding (*sense*) gene, and play various, important regulatory roles by forming extensive base-pairing interactions with the corresponding sense RNA [16], can be revealed in metatranscriptomic sequences. Antisense RNAs range in size from tens to thousands of nucleotides, complementing with part of a gene, a complete gene or a group of genes [17,18]. Although asRNAs were first observed in bacteria more than 30 years ago [19], most studies of asRNAs in bacteria are rather recent, and have been applied mainly to single bacterial species, including *Chlamydia trachomatis *[20] and *Escherichia coli *[21]. Metatranscriptomics research is creating an unprecedented opportunity to gain knowledge about the gene regulation for the vast majority of uncultured microbial species.

In this paper, we present a gene finding software tool (TransGeneScan) specifically designed for metatranscriptomic sequences that addresses all features described above. TransGeneScan incorporates strand-specific hidden states, representing coding sequences in sense and antisense strands in a Hidden Markov Model (HMM), which is different from the HMM that we used in FragGeneScan [22]. As a result, TransGeneScan can predict a sense transcript containing one or multiple genes (in an operon) or an antisense transcript. We note that TransGeneScan inherits the advantage of FragGeneScan in that the parameters of the HMM can be computed based on the GC% of the input sequences and therefore no trainings on specific data sets are needed, a desirable feature for gene finding in metagenomic/metatranscriptomic sequences. We tested TransGeneScan on a mock metatranscriptomic data set containing 3 bacterial genomes, and compared its performance with metagenomic gene finders MetaGeneMark [23] and FragGeneScan [22], as well as Glimmer [24] and GeneMark [25], gene finders trained for each specific bacterial genome (assuming their presences in the metatranscriptome are known). The results showed that TranGeneScan performs much more accurately than metagenomic gene finders on metatranscriptomic sequences, and achieves comparable or even higher accuracy than gene finders for microbial genomes. These results imply, with the assistance of metatranscriptomic sequencing, we can obtain a broad and precise picture about the genes (and their functions) in a microbial community.

## Methods

TransGeneScan takes as input a set of transcript sequences, and reports the annotation of these sequences, either sense RNA (including mRNAs that encode one or more protein coding genes, and RNAs that don't contain coding regions), or asRNAs with regulatory functions. It is built upon a Hidden Markov model that considers each input transcript sequence as an observation sequence, and computes the most likely hidden sequence, representing the annotation of the sequence. The metatranscriptomic sequences acquired by using a stranded RNA-seq protocol will be first assembled into transcripts by a reference-mapping approach (e.g., through mapping the reads to reference bacterial genomes or corresponding metagenomes) or by a *de novo *assembly approach, e.g., by using Velvet [26], Trinity [27] or Oases [28]. We tested TransGeneScan on a metatranscriptomic data set acquired from a mock community consisting of three bacterial species: *Escherichia coli, Rhodobacter sphaeroides*, and *Prochlorococcus marinus *[15]. To evaluate the performance of TransGeneScan, we focused on the accuracy of the protein-coding genes on the predicted mRNA transcripts, where the currently annotated genes in each of the three genomes are considered to be true positives. We also analyzed the asRNAs and operons predicted by TransGeneScan. Below, we describe these methods in details.

### Transcript assembly

In this study, we adopt an *ad hoc *reference-based assembly algorithm for reconstructing transcript sequences from metatranscriptomic data. Transcriptome assemblers are available for processing RNA-seq data, including reference-based transcriptome assemblers such as Cufflinks [29] and *de novo *assemblers such as Velvet [26], Trinity [27] and Oases [28]. These algorithms were designed for eukaryotic transcriptome assembly, and thus focused on the challenge of reconstructing alternative splicing forms. On the one hand, the assembly of bacterial transcriptomes might be less challenging because of the lack of splicing; but specific issues like overlapping transcripts and alternative operon structures may complicate the problem [30]. As a result, fine-tuned algorithms may be needed for metatranscriptome assembly. Nevertheless, we stress that implementation of a metatranscriptome assembler is beyond the scope of this paper, and hence, we adopt a simple approach to this problem, as described below.

Given a set of RNA-seq sequences, we first align them onto reference genomes by using BWA [31]. To obtain the start and end positions of transcripts from the mapped reads, we partition the reads into two sets using SAMtools [32]: the first set contains reads contributed by transcripts transcribed from the positive strand of the reference genome (i.e., *positively-transcribed *reads), and the second set contains reads contributed by those transcribed from the negative strand (i.e., *negatively-transcribed *reads). Because the stranded RNA-seq protocol is used, this partitioning can be easily achieved using the SAMtools flag-filters based on the rules as shown in Supplementary Table S1 (see Additional file 1). In the next step, for each set, we mark regions in the reference genome supported by at least one read from the respective set. Then, we extract contiguous marked regions that are *≥ *120 base pairs long. These sequences are considered as potential transcripts and input to the gene prediction programs. Since we know that the transcripts assembled from the second set of reads correspond to transcripts from the negative strand, these transcripts are converted into their reverse complements before giving as input to the prediction programs. The gene annotations for the transcripts are obtained from the corresponding annotations for the reference genome, which are downloaded from Genbank (IDs: *E. coli*: NC_000913.3, *P. marinus*: NC_005072.1 and *R. sphaeroides*: NC_007493.2). We used the reference genomes to achieve a well-assembled set of transcripts, for the purpose of evaluating the gene-prediction accuracy in metatranscriptomic data. In practice, complete reference genomes may not be available for metatranscriptome studies. In this case, one can use transcripts assembled by *de novo *assembly algorithms (such as Velvet [26], Trinity [27] or Oases [28]) as input to TransGeneScan.

### Hidden Markov Model for gene finding

**Figure 1:**
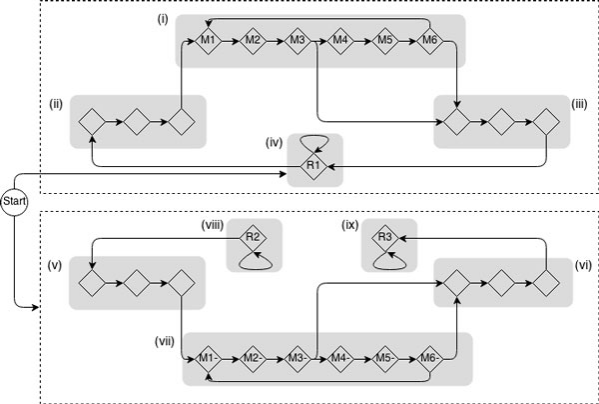
**The Hidden Markov Model employed in TransGeneScan**. The model consists of 9 super-states in two modules, 4 for the sense (coding) strand (top module), representing coding regions (i), start codons (ii), stop codons (iii) and un-translated regions (iv), respectively; and 5 for the antisense strand (bottom module), representing start codons (v), stop codons (vi), coding regions (vii), and un-translate regions (viii and ix), respectively. The un-translated regions in the antisense strand are represented as two distinct states, one for the 5' un-translated region and one for the 3' un-translated region to prohibit the transition from the coding regions in one gene to those on another (because antisense transcripts are often a part of gene in the opposite strand). Furthermore, an idle start state is used to ensure that the annotation (hidden state) sequence can only initiate from the un-translated regions in positive strand (but can initiate from any state in the negative strand). The transition from the hidden states in one strand to the states in another strand is prohibited. Each of the two super-states for coding regions (i and vii) consists of six consecutive match states (M1 to M6, and M1- to M6-, respectively) represented by diamonds, which collectively correspond to a six-periodic inhomogeneous HMM. Comparing to the HMM used in FragGeneScan [22], this model does not contain the insertion and deletion states, based on the assumption that the assembled transcripts from metatranscriptomic sequences contain no frameshift errors.

In TransGeneScan, we extended the Hidden Markov Model (HMM) used in FragGeneScan to the gene finding in metatranscriptomic sequences. FragGeneScan HMM incorporates codon usage bias, sequencing error models and start/stop codon patterns in a unified model for gene finding in short, error-prone metagenomic sequences [22]. The model parameters (e.g., the transition and emission probabilities) were not learned from training data, but were estimated by using a linear regression to the GC% of the input genomic sequences. We modified the FragGeneScan by removing the frameshift states (based on the assumption that the assembled transcript sequences contain no frameshift errors), and incorporating the strand specificity of the transcript. As shown in Figure 1, the HMM employed in TransGeneScan consists of 9 super-states in two modules: 4 super-states in the sense (coding) strand module, representing coding regions, start codons, stop codons and un-translated regions, respectively; and 5 super-states in the antisense strand module, representing start codons, stop codons, coding regions, and un-translated regions, respectively. The un-translated regions in the antisense strand are represented as two distinct states, one for the 5' un-translated region and one for the 3' un-translated region to prohibit the transition from the coding regions in one gene to those in another (because antisense transcripts often overlap with only one gene). Furthermore, an idle start state is used to ensure that the annotation (hidden state) sequence can only initiate from the un-translated regions in positive strand (but can initiate from any state in the negative strand). We removed transitions from the forward strand to the backward strand and vice versa making the top and the bottom half of the model mutually exclusive. Each of the two super-states for coding regions consists of six consecutive match states (M1 to M6, and M1- to M6-, respectively) represented by diamonds, allowing different parameters to be used for each position in a di-codon, which collectively model the codon bias in coding regions. We used the same regression models as used in FragGeneScan to obtain transition and emission probabilities for the match states [22].

To annotate transcript sequences, the Viterbi algorithm is used to obtain the most likely path of hidden states that generates the input nucleotide sequence. In this study, we output coding sequences of length greater than 120 base pairs that start in a start state (start codon) and end in a stop state (stop codon). A length of 120 bps is chosen to ensure the transcript contains at least two overlapping reads, since the length of Illumina reads is about 100 bps. Genes predicted in the strand opposite to the native strand of the transcript (denoted as the negative strand) are reported as antisense transcripts. While more than one open reading frames are allowed to be predicted in the native strand of a transcript (i.e., corresponding to an operon), at most one is allowed in the antisense strand.

TransGeneScan is implemented using C and Perl, and is available as open-source software on SourceForge at https://sourceforge.net/projects/transgenescan/.

### Performance evaluation and comparison

The performance of gene prediction is measured in terms of sensitivity, precision and accuracy. Sensitivity (Sn) is computed as the ratio of true positives versus all annotated genes and precision (Pr) is the ratio of true positives versus all predicted genes. The accuracy is measured using the F-score defined by,

(1) $$F=2\cdot \frac{Pr\cdot Sn}{Pr+Sn}$$

The performance of TransGeneScan is compared with GeneMark, Glimmer, MetaGeneMark and FragGeneScan. The parameters used for these programs are shown in Table 1. All the programs are given the same input transcript sequences. Partial gene predictions are removed from the output of all programs for the purpose of performance evaluation.

**Table 1:** Command lines and parameters used for the programs in the benchmarking.

GeneMark:
$ gmsn.pl *−−*prok *−−*format GFF *<*input*>*
Glimmer:
$ build *−*icm *−*r run1.icm *< <*coding*−*sequences*>*
$ glimmer3 *−*o50 *−*g110 *−*t30 *<*input*>*run1.icm run1

MetaGeneMark:
$ gmhmmp *−*s . *− *f G *−*m MetaGeneMark v1 . mod *−*o mgm.gff *<*input*>*

FragGeneScan:
$ run_FragGeneScan.pl *−*genome=*<*input*> −*out=Frag
*−*complete=1 *− *train =complete

## Results and discussion

We analyzed RNA-seq reads from a mock bacterial community containing three bacterial species [15]: *Escherichia coli *[GenBank:NC_000913.3], *Prochlorococcus marinus *[GenBank: NC_005072.1] and *Rhodobacter sphaeroides *[GenBank:NC_007493.2]. The set of raw RNA reads (paired-end 101 bps reads acquired by using Illumina sequencer) were downloaded from Short Reads Archive (SRA ID: SRR442380), which contains 9,668,044 paired-end reads of 976.5 Mbps with the average insertsize of 300 bps. The reads were mapped to the three reference genomes by using BWA [31], and a total of 5,278,699, 2,439,476 and 1,113,601 reads can be mapped to the genomes of *E. coli, P. marinus *and *R. sphaeroides*, respectively. These mapped reads were further assembled into the transcripts representing genomic sequences continuously covered by mapped reads in each genome (see Methods for details). These transcripts were provided as input to TransGeneScan, which classified each transcript as a sense transcript or an antisense transcript. For sense transcripts, TransGeneScan also reported the protein-coding genes in them.

### Accuracy of protein-coding gene prediction

We first evaluate the prediction accuracy of TransGeneScan on protein-coding genes. The performance is compared with two metagenomic gene finders MetaGeneMark and FragGeneScan, and two gene finders for microbial genomes, Glimmer and GeneMark. The default parameters were used for TransGeneScan, MetaGeneMark and FragGeneScan, and thus are independent of the input sequences used here for performance evaluation. Notably, the parameter used in TransGeneScan for estimating transition and emission probabilities are inherited from FragGeneScan [22], which were trained on a set of genes in bacterial genomes with various GC-content [22]. The parameters of Glimmer and GeneMark were self-trained on protein-coding genes in each respective bacterial genome, and as a result, different model parameters might be used in the gene prediction on transcripts from each of the three bacterial genomes. We note that, in practice of metatranscriptomic gene prediction, the bacterial genomes from which some transcripts are transcribed may not be known; as a result, Glimmer and GeneMark may not perform as well as reported here.

The performance of these five programs were measured on sensitivity, precision and accuracy (see Methods), as shown in Table 2. A gene annotated in a respective reference genome is considered to be positive if it is fully contained in an assembled transcript. A total of 2,171, 621 and 1,184 genes can be recovered in the assembled transcripts of the metatranscriptome sequences in the bacteria *E. coli, P. marinus *and *R. sphaeroides*, respectively. A predicted gene is considered to be *true positive *if it completely overlaps with (or overlaps with at least 80% of) an annotated gene in the corresponding frame in the respective reference genome. The rest of predicted genes are counted as *false positives*. We found that a significant proportion of false positives correspond to predictions in partial genic regions at the 5' end of the transcripts. For these genes, the stop codon is predicted correctly in the correct frame; but the start codon is picked from the middle of the gene since the transcript does not cover the entire gene. Because these genes can neither be counted as true positives nor as false positives, they are excluded from the performance evaluation for each of the five programs. A gene is considered to be *false negative *if it is covered by a transcript, but does not completely overlap with (or overlap with at least 80% of) a predicted gene in the corresponding frame.

**Table 2:** Comparison of performance measures (TP - True Positives, Sn - Sensitivity, Pr - Precision and Ac - Accuracy) between GeneMark, Glimmer, MetaGeneMark (MGM), FragGeneScan (FGS) and TransGeneScan (TGS).

Organisms		GeneMark	Glimmer	MGM	FGS	TGS
E. coli (2171*∗*)	Predicted	2039	2169	1961	1941	2159

Completely	TP	1805	1695	1642	1678	**1889**
Overlap	Sn	83.14	78.07	75.63	77.29	**87.01**
	Pr	97.67	95.71	97.56	96.00	**97.82**
	Ac	89.82	86.00	85.21	85.63	**92.10**

80%	TP	1996	2093	1920	1871	**2117**
Overlap	Sn	91.94	96.41	88.44	86.18	**97.51**
	Pr	97.89	96.50	97.91	96.39	**98.05**
	Ac	94.82	96.45	92.93	91.00	**97.78**

P. marinus (621*∗*)	Predicted	631	698	592	578	571

Completely	TP	488	**527**	482	456	501
Overlap	Sn	78.58	**84.86**	77.62	73.43	80.68
	Pr	83.85	83.52	88.93	85.23	**95.98**
	Ac	81.13	84.19	82.89	78.89	**87.66**

80%	TP	537	**595**	532	499	550
Overlap	Sn	86.47	**95.81**	85.67	80.35	88.57
	Pr	85.10	85.24	89.86	86.33	**96.32**
	Ac	85.78	90.22	87.72	83.24	**92.28**

R. sphaeroides (1184*∗*)	Predicted	1078	1121	1024	1026	1165

Completely	TP	899	891	897	879	**1011**
Overlap	Sn	75.93	75.25	75.76	74.24	**85.39**
	Pr	98.04	97.38	**98.36**	97.88	97.87
	Ac	85.58	84.90	85.59	84.44	**91.20**

80%	TP	1060	1097	1009	1007	**1143**
Overlap	Sn	89.53	92.65	85.22	85.05	**96.54**
	Pr	98.33	97.86	**98.54**	98.15	98.11
	Ac	93.72	95.18	91.39	91.13	**97.32**

^∗^The numbers of *positive *genes recovered in the assembled transcripts.

From the results, we observe that TransGeneScan performs significantly better than metagenomic gene finders (MetaGeneMark and FragGeneScan), especially with respect to sensitivity. It performs comparable to GeneMark and Glimmer with some tradeoff between sensitivity and precision (e.g, in the case of *E. coli *and *R. sphaeroides*, TransGeneScan achieves higher sensitivity, while in the case of *P. marinus*, it achieves better precision). The overall accuracy of TransGeneScan is better than that of GeneMark and Glimmer. It was observed that gene prediction accuracy in metagenomic sequences is typically lower than that in microbial genomes, because the average model parameters are used for different bacterial genomes potentially present in the input data [23,22]. Our results, however, indicate that with the assistance of metatranscriptomic data, bacterial genes can be predicted accurately in metagenomes, with comparable or even higher prediction accuracy (i.e., *>*90%) than that in bacterial genomic sequences (i.e., typically 80% to 90%). We attribute this improvement to the TransGeneScanentary signals in the metatranscriptomic sequences: 1) the boundary of transcripts help to define the appropriate reading frame as well as the start codon; and 2) the stranded data help to distinguish the true coding strand. We stress that we used the reference genome here only for evaluation purpose, i.e., by considering annotated genes in the reference as the golden standard. In practice, one can use TransGeneScan to directly predict genes in assembled transcripts from metatranscriptomic sequences without additional training, and the prediction accuracy should not be substantially worse than the results described here. Finally, gene prediction in a particular metatranscriptomic data set may not cover all genes encoded in the community (e.g., in the mock data set used here, 2,159 or 52% genes in *E. coli *can be recovered from assembled transcript), because 1) only a fraction of genes are transcribed, and 2) due to the sequencing depth, some transcribed genes with low abundances in the sample may not be fully recovered. Therefore, multiple metatranscriptomic studies of the same community are needed to achieve a comprehensive annotation of the genes in the community.

### Predicted antisense transcripts

In addition to sense transcripts, TransGeneScan predicted a substantial number of antisense transcripts: among 5,999, 3,027 and 3,173 transcripts predicted in *E. coli, P. marinus *and *R. sphaeroides*, 2,086 (34.8%), 1,094 (36.1%) and 490 (15.6%) were predicted as putative antisense transcripts, respectively. A majority of these putative antisense transcripts (2,681 out of 3,670; 73.1%) overlaps partially with one real gene (as annotated in the reference genome) in the opposite strand, while do not overlap with any gene in the same strand; 1,585 (76.0%), 932 (85.2%) and 164 (33.5%) of those cases are in *E. coli, P. marinus *and *R. sphaeroides*, respectively. These predicted transcripts are likely true positives, although the remaining predictions are not necessarily false. Only very few (0.52%, 2.65% and 2.45%) predicted antisense transcripts contain complete annotated genes (11, 29 and 12 in *E. coli, P. marinus *and *R. sphaeroides*, respectively), which are likely mRNAs instead of asRNAs, and thus are false positives. The numbers of asRNAs reported for different bacteria vary extensively, but hundreds and even thousands have been suggested in some species [33]. Our analysis suggests a widespread antisense transcription in *E. coli*, which is consistent with the previous systematic studies that report thousands of asRNAs in this species [21,34]. We also found prevalent asRNAs in the less studied bacterial species (*P. marinus *and *R. sphaeroides*). We report these predicted asRNAs in our website (http://omics.informatics.indiana.edu/mg/TransGeneScan), which will provide important resources for further studies of the gene regulation in these species.

### Predicted operons

TransGeneScan can predict more than one gene in a given transcript, which indicates a putative operon structure (i.e., multiple genes transcribed together in the same transcript). We observed a total of 512, 158 and 321 putative operons predicted in *E. coli, P. marinus *and *R. sphaeroides*, respectively. We compared the 512 operons predicted in *E. coli *with the known operons curated in RegulonDB [35]. Out of 512 predicted putative operons, 445 (86.9%) matched with at least 80% overlap of those in RegulonDB, and 144 (28.1%) matched completely with known operons. Among the remaining 67 predicted operons, 65 were fully contained within the operons in the RegulonDB predominantly with at least 30% overlap. These cases may indicate incomplete coverage of the transcripts containing these operons in the data set due to low sequencing depth, or potential alternative operon transcription in the experiment. There were two predicted operons that span across multiple known operons: one contains the genes *ygbK *and *ygbL*, spanning two operons ygbJK (containing genes *ygbJ *and *ygbK*), and ygbLM (containing genes *ygbL *and *ygbM*) in regulonDB; the other one contains the genes *yjeM *(partial), *yjeN *and *yjeO*, spanning the two operons yjeM (containing gene *yjeM*) and yjeNO (containing genes *yjeN *and *yjeO*). The intergenic distances in the known operons are -3 (overlapping genes), 5 and -3 between the pairs of genes of ygbJK, ygbLM and yjeNO, respectively, whereas in the two predicted operons, the intergenic distances are 92 (between ygbKL) and 52 (between ybeMN), respectively. These two cases may represent novel alternative operon structures, or artificial transcripts that merge two overlapping transcripts (we note that computational methods have been developed recently to detect overlapping transcripts in bacterial RNA-seq data [30]). Nevertheless, our results showed that most of the putative operons predicted in *E. coli *by TransGeneScan agreed with the ones collected in RegulonDB. Hence, TransGeneScan prediction on metatranscriptomic data may provide useful information to detect active operons in a microbial community. The full list of operons in all three genomes are shown in our website (http://omics.informatics.indiana.edu/mg/TransGeneScan).

## Conclusion

In this paper, we present TransGeneScan, a software tool specifically designed for finding genes in metatranscriptomic sequences. TransGeneScan can predict protein-coding genes as well as antisense RNAs solely from metatranscriptomic sequences without additional training. The testing results showed that TranGeneScan achieves comparable or even higher accuracy than gene finders for individual microbial genomes, implying that, with the assistance of metatranscriptomic sequencing, we can predict accurately the genes in a microbial community, and thus reveal a precise picture of its functional properties.

## List of abbreviations

HMP: Human Microbiome Project; T2D: Type II Diabetes; NGS: Next generation sequencing; asRNA: antisense RNA; HMM: Hidden Markov Model; Sn: Sensitivity; Pr: Precision: Ac: Accuracy; TP: True positives; MGM: MetaGeneMark; FGS: FragGeneScan; TGS: TransGeneScan.

## Competing interests

The authors declare that they have no competing interests.

## Authors' contributions

HT, YY and WMI designed the study. WMI contributed tools for the analysis. WMI, HT and YY analyzed the data, and wrote the paper.

## Supplementary Material

Additional file 1 **Supplementary tables**. PDF file containing Supplementary Table S1. Click here for file

## Declarations

Publication of this article was supported by National Science Foundation under grant number DBI-0845685 and RECOMB conference.

This article has been published as part of BMC Bioinformatics Supplement: Proceedings of the Fourth Annual RECOMB Satellite Workshop on Massively Parallel Sequencing (RECOMB-seq 2014).

This article has been published as part of BMC Bioinformatics Volume 15 Supplement 9, 2014: Proceedings of the Fourth Annual RECOMB Satellite Workshop on Massively Parallel Sequencing (RECOMB-Seq 2014). The full contents of the supplement are available online at http://www.biomedcentral.com/bmcbioinformatics/supplements/15/S9.
