# Supplementary material for: Gene finding in metatranscriptomic sequences
Source: BMC Bioinformatics. 2014 Sep 10;15(Suppl 9):S8. doi: 10.1186/1471-2105-15-S9-S8 (PMC4168707; doi:10.1186/1471-2105-15-S9-S8)
Supplement: Additional file 1 — Supplementary tables. PDF file containing Supplementary Table S1. [file 1471-2105-15-S9-S8-S1.pdf]

Supplementary Tables

Table S1: SAM flags used to partition the stranded RNA-Seq reads into positive-transcribed and negative transcribed reads.

Strand of the transcript	SAMtool flags (Breakdown)	Explanation
Positive	83 (1+2+16+64)	A read that is the 1st read in a pair, and is mapped to negative strand while its mate is mapped to positive strand.
	163 (1+2+32+128)	A read that is the 2nd read in a pair, and is mapped to positive strand while its mate is mapped to negative strand.
Negative	99 (1+2+32+64)	A read that is the 1st read in a pair, and is mapped to positive strand while its mate is mapped to negative strand.
	147 (1+2+16+128)	A read that is the 2nd read in a pair, and is mapped to negative strand while its mate is mapped to positive strand.
